# Genome sequence analysis of *Robertmurraya* sp. GLU-23 isolated from coal seam formation water

**DOI:** 10.1128/mra.01187-25

**Published:** 2026-03-20

**Authors:** Yuhang Zhang, Yuqi Wang, Li Fu, Juanli Yun

**Affiliations:** 1 School of Environmental Science and Engineering, Shaanxi University of Science and Technology 74618, Xi’an, China; 2 Key Laboratory of Development and Application of Rural Renewable Energy, Ministry of Agriculture and Rural Affairs, Biogas Institute of Ministry of Agriculture and Rural Affairs, Chengdu, China; Wellesley College, Wellesley, Massachusetts, USA

**Keywords:** coal seam formation water, isolation, *Robertmurraya*, polycyclic aromatic hydrocarbons (PAHs), coal degradation bacteria

## Abstract

This report presents the genome sequence of *Robertmurraya* sp. GLU-23, a potential coal-degrading bacterium isolated from coal seam producing water in Hancheng, Shaanxi, China. The draft genome, with 5,059,906 bp and 37.87% GC content, contains genes for polycyclic aromatic hydrocarbon biodegradation, suggesting its potential for coal degradation.

## ANNOUNCEMENT

A coal seam is a deep underground ecosystem that is driven by organic matter from coal and is rich in methane. It supports a highly specialized and diverse microbial community compared to other environments (1). Members of *Robertmurraya* were previously classified as species belonging to *Bacillus* and have been recently recognized as *Robertmurraya* according to their phylogenetic heterogeneity (2). However, their role in coal seams is still unknown.

*Robertmurraya* sp. GLU-23 was isolated from a coal seam formation water (35.47625^o^N, 110.45081^o^E) sample in Hancheng, Shaanxi, China. Five milliliters of coal seam formation water was inoculated into a 45 mL inorganic salt medium containing KH_2_PO_4_, MgSO_4_·7H_2_O, NaCl, CaCl_2_·2H_2_O, NaHCO_3_, resazurin, trace elements, vitamins (3), and Na_2_S (1 mM) in an anaerobic bottle, using 20 mM glucose as the sole carbon source. The anaerobic bottle was incubated at 35°C in the dark for 60 days. After incubation, 100 μL of enrichment was used for Hungate tube isolation (using the same medium as enrichment, with 1.5% agar added). A single colony was picked and cultured in a 5 mL anaerobic tube containing liquid medium (same as enrichment medium) at 35°C for 10 days. Genomic DNA was extracted from 3 mL of liquid culture using the SPINeasy DNA Pro Kit for Soil (Guangzhou, China) following the manufacturer’s protocol. The 16S rRNA gene was amplified using TSINGKE TSE008 2× T5 Super PCR Mix (Tsingke Biotechnology, Nanjing, China) with 27F/1492R (5′-AGAGTTTGATCCTGGCTCAG-3′/5′-TACGGCTACCTTGTTACGACTT-3′) primer set according to the manufacturer’s instructions (4). PCR products were sequenced using Sanger sequencing technology (Tsingke Biotechnology) and blasted at Ezbiocloud (https://www.ezbiocloud.net/); the closest relative is *Robertmurraya korlensis* (EU603328), with a similarity of 91.86%.

Genomic sequencing was performed on the Illumina PE150 NovaSeq X Plus platform (Majorbio, Shanghai, China). Briefly, the library was prepared using the ALFA-SEQ DNA Library Prep Kit (cat. no. NDI001E; FINDROP, China). Sequence data were trimmed using Fastp (v.0.23.2) (5), assembled using SPAdes (v.4.0.0) (6) and GapCloser (v.1.12) (7). The completeness and contamination were evaluated through CheckM (v.1.1.2) (8). Taxonomic classification was performed via GTDB-Tk (v.2.4.0, r220) (9). The assembled genome was then annotated using the National Center for Biotechnology Information Prokaryotic Genome Annotation Pipeline (v.6.9) (10). All the software uses default parameters unless specified. The detailed annotation results can be found in the NCBI database under accession number JBRKCV000000000. The genomic features of *Robertmurraya* sp. GLU-23 are summarized in Table 1. The average nucleotide identity (ANI) between *Robertmurraya* sp. GLU-23 and *R. korlensis* (GCA_001591645.1) was calculated using fastANI (v.1.34) (11). The ANI value is 87.16% (Table 1).

**TABLE 1:** Genomic statistics of *Robertmurraya* sp. GLU-23

Parameter	Data
Sequence Read Archive accession number	SRR35655281
BioSample accession number	SAMN52028738
WGS accession number	JBRKCV000000000
No. of raw reads	6,362,308
Completeness (%)	99.45
Contamination (%)	4.11
G+C content (%)	37.87
N50 (bp)	244,017
No. of contigs	60
Genome size (bp)	5,059,906
No. of CDSs	4,913
No. of tRNAs	120
No. of mRNAs	39
ANI to *Robertmurraya korlensis* (GCA_001591645.1) (%)	87.16

Function annotation of *Robertmurraya* sp. GLU-23 indicates a putative role in PAH degradation. The genome encodes key enzymes, including phenanthrene-degrading dioxygenase (*nidA*) and phthalate 3,4-dioxygenase (*phtA*), which facilitate the degradation of typical substrates in coal seams (12). This announcement highlights the broad potential of this strain for bioremediation and coal bed methane production.

## Data Availability

The raw Illumina reads for *Robertmurraya* sp. GLU-23 have been deposited in the Sequence Read Archive under accession number SRR35655281. This Whole Genome Shotgun project has been deposited at GenBank under accession number JBRKCV000000000, and the version described in this paper is version JBRKCV000000000.1.
